# A Case of Membranous Nephropathy Hypothesized to be Associated With COVID-19 Vaccine

**DOI:** 10.7759/cureus.24245

**Published:** 2022-04-18

**Authors:** Wahida Rashid, Heba Mousa, Jahanzeb Khan, Fakhar Ijaz, Gerry D Ezell

**Affiliations:** 1 Internal Medicine, Baptist Health - University of Arkansas for Medical Sciences, North Little Rock, USA; 2 Nephrology, Baptist Health North Little Rock, North Little Rock, USA

**Keywords:** interstitial nephritis, pla2r, formaldehyde, covid-19 vaccine, membranous glomerulonephritis

## Abstract

A 56-year-old male patient with a medical history of essential hypertension was referred to the emergency room after he was found to have a serum creatinine level of 13 mg/dL at his primary care physician’s office. The patient reported that he had developed a coronavirus disease 2019 (COVID-19)-like infection six months prior that was not confirmed. Two months later, he started to notice dyspnea on exertion and bilateral lower limb swelling and was started on furosemide. He received the first dose of the Moderna COVID-19 vaccine a month before the presentation but did not receive the second dose. Subsequently, his lower limb swelling and exertional dyspnea started worsening. He denied any new medication, dysuria, oliguria, hematuria, fever, or any other symptoms. Initial evaluation was consistent with kidney failure. Hypocalcemia and hyperphosphatemia were noted, along with medical renal disease on renal ultrasound. Eosinophils and nephrotic-range proteinuria were found in the urine. His serum phospholipase A2 receptor (PLA2R) antibodies were positive. A renal biopsy showed membranous glomerulonephritis with moderate segmental sclerosis, as well as tubulointerstitial fibrosis with neutrophils, consistent with acute interstitial nephritis. Positive staining for PLA2R in the glomerular deposits suggested primary membranous nephropathy (MN). He was treated with prednisone first, and when the kidney biopsy was conclusive for membranous glomerulopathy, he was started on rituximab. On admission, he received hemodialysis intermittently, but this was stopped a month after discharge as his renal function normalized. Recently, there have been numerous cases reported with new onset of glomerular disease after receiving the COVID-19 vaccine. Further studies of vaccinated patients are needed to determine whether the severe acute respiratory syndrome coronavirus 2 virus vaccination is associated with a higher risk of MN and to identify potential predisposing factors and mechanisms of kidney injury in patients in whom it occurs.

## Introduction

Membranous nephropathy (MN) is a common cause of nephrotic syndrome globally. Primary MN accounts for about two-thirds of all MN cases. In the remaining cases, MN may be secondary to infections, influenza vaccinations [1], drugs (non-steroidal anti-inflammatory drugs (NSAIDS), gold, captopril, penicillamine), cancer, or autoimmune diseases [2]. Here, we describe a patient who was diagnosed with MN after administration of the first dose of the Moderna coronavirus disease 2019 (COVID-19) vaccine with no other apparent etiology except a recent history of COVID-19-like symptoms which were not confirmed. This case suggests an association between the severe acute respiratory syndrome coronavirus 2 (SARS-CoV-2) Moderna vaccine and phospholipase A2 receptor (PLA2R)-positive MN.

## Case presentation

A 56-year-old man, with a two-year history of hypertension, presented to the emergency department (ED) with concerns over abnormal laboratory results obtained by his primary care physician (PCP) as part of a routine office visit for medication refills. He was unexpectedly found to have elevated creatinine, hypocalcemia, and anemia. At the time of his ED visit, he complained of fatigue, exertional dyspnea, and new lower urinary tract symptoms. He had been taking aspirin for years and used NSAIDs occasionally. The only other medicine he was taking was lisinopril-hydrochlorothiazide (20-25 mg) and he was compliant with it. He had experienced COVID-19-like symptoms (cough, sore throat, malaise, loss of taste and smell) six months prior but was never tested for COVID-19. Two months after these symptoms, he noticed the onset of bilateral lower extremity edema for which his PCP prescribed furosemide. He did not have a history of congestive heart failure. He reported no symptoms of flank pain, abdominal pain, previous lower urinary tract symptoms, or systemic symptoms such as fever, chills, nausea, vomiting, diarrhea, or weight loss. He did not report chest pain, hematuria, or any change in his ability to taste and smell. He had received the first dose of the COVID-19 mRNA vaccine by Moderna one month prior to the current hospital admission but had not received the second dose. He worked as a mortician and had been employed in this occupation for approximately 30 years. He had frequent occupational exposure to formaldehyde and ethanol.

On presentation, the patient was afebrile. He was hypertensive (blood pressure of 171/128 mmHg), with the remainder of his vital signs normal. His electrocardiogram and chest X-ray were normal. Abnormal laboratory findings included serum creatinine 13.96 mg/dL and blood urea nitrogen 69 mg/dL. Furthermore, urine analysis showed 2+ protein and 2+ blood, complete blood count showed hemoglobin 6.9 mg/dL, hematocrit 32.1%, serum iron 36 µg/dL, ferritin 578.3 ng/mL, total iron-binding capacity 169 µg/dL, serum calcium 6.9 mg/dL, vitamin D 12.4 ng/mL, phosphate 5.5 mg/dL, parathyroid hormone 349 pg/mL hemoglobin A1c 4.8%, urine total protein 16,989 mg/L, and urine creatinine 138.8 mg/dL; moreover, eosinophils were detected in the urine. Other pertinent laboratory results included positive findings for serum PLA2R antibodies, enzyme-linked immunosorbent assay 265, and PLA2R antibodies on indirect fluorescent antibody assay. Serum albumin was low at 2.2 g/dL, and lipids were unremarkable. Other testing, including serum protein electrophoresis, prostate-specific antigen, hepatitis panel, and autoimmune screening panel, were all negative. Imaging did not show any evidence of malignancy. His renal ultrasound showed bilateral medical renal disease, a simple right renal cyst, and an enlarged prostate. There was increased echogenicity of the renal cortex and no hydronephrosis. Vascular imaging showed no evidence of thrombosis.

**Figure 1:**
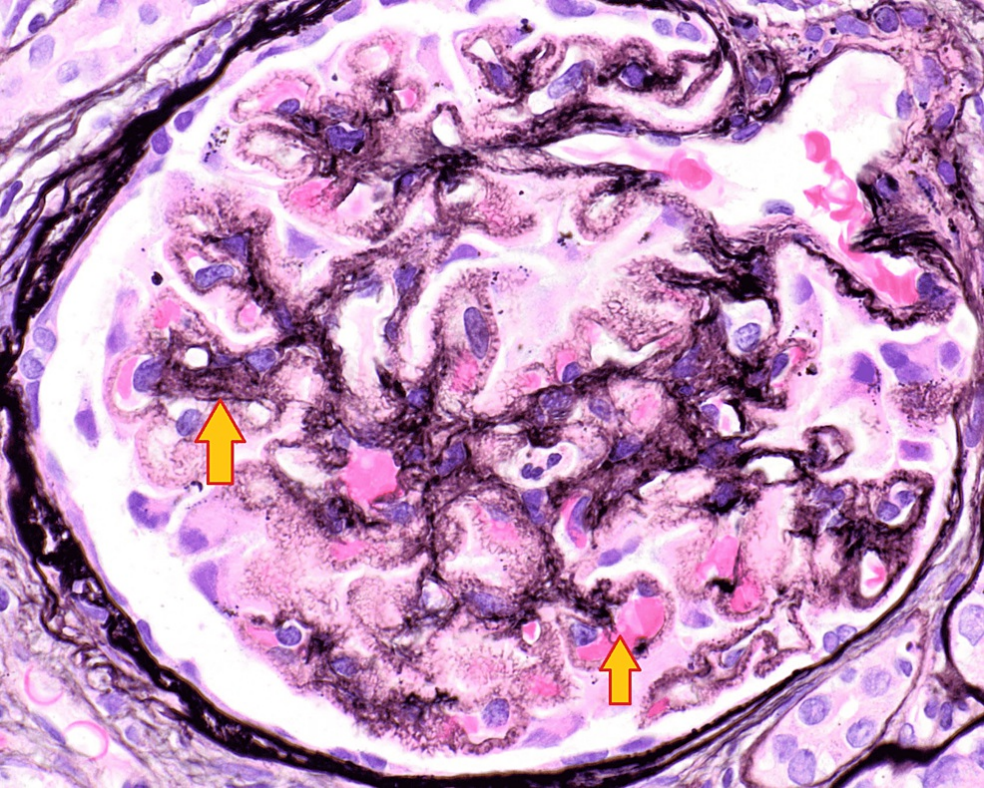
Glomerular membrane thickening. The arrows show glomerular membrane thickening.

**Figure 2:**
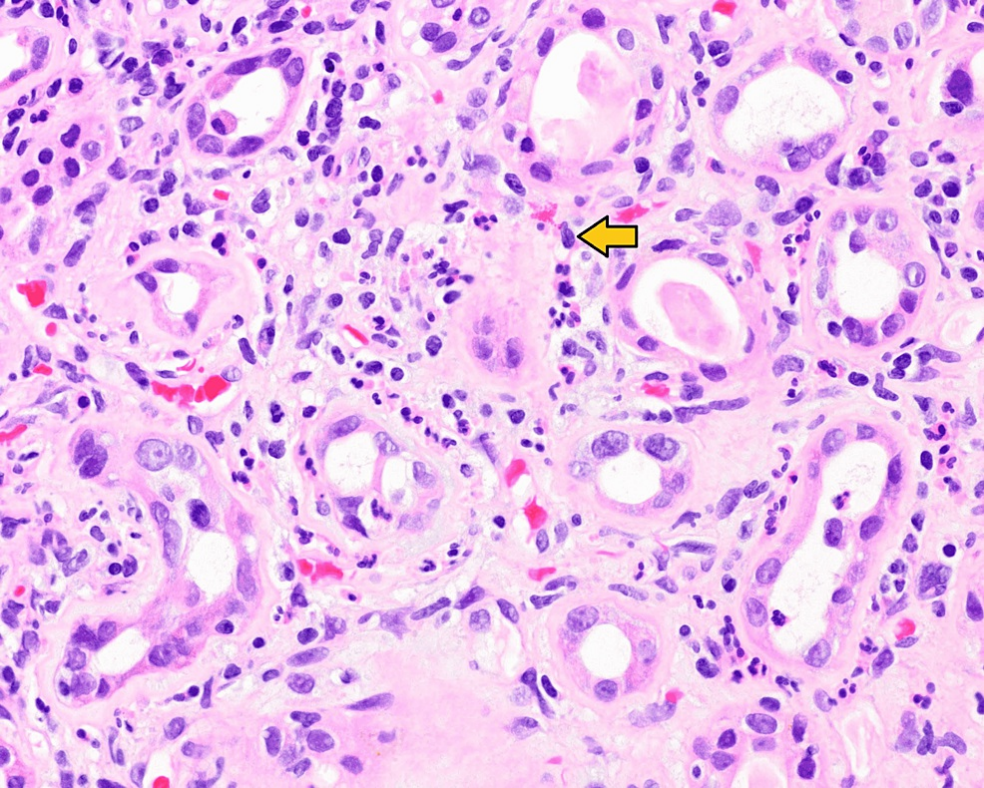
Neutrophilic interstitial inflammation. The arrow shown indicates neutrophilic interstitial inflammation.

**Figure 3:**
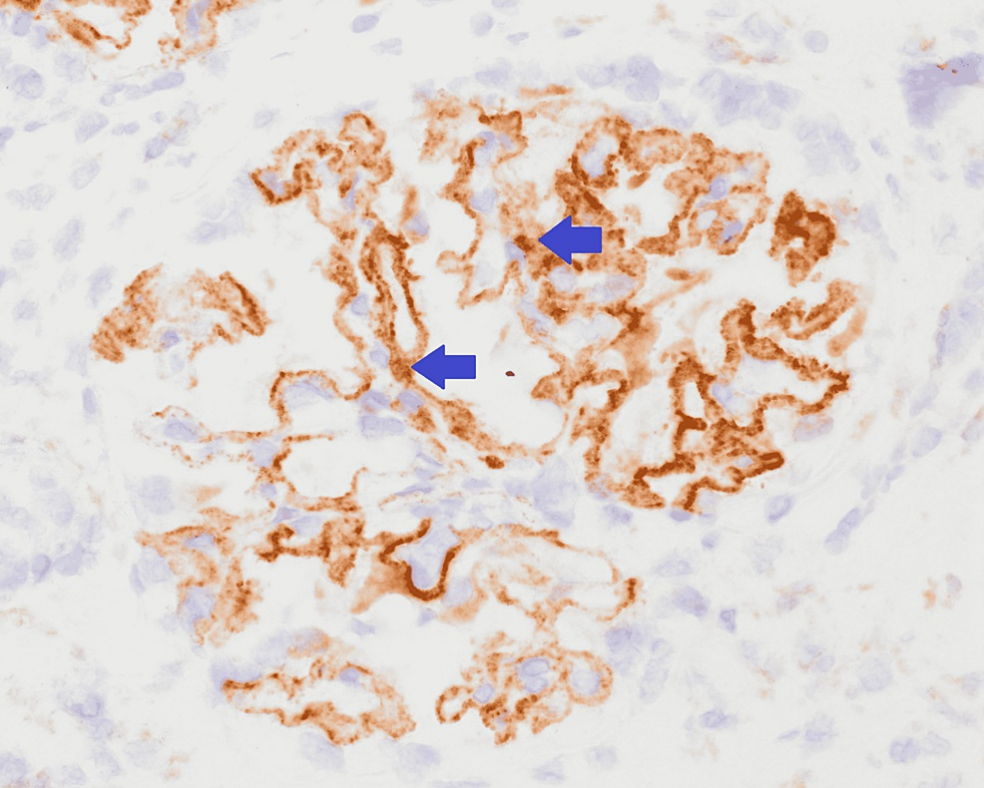
Glomerulus with PLA2R-positive antibodies. Positive staining for PLA2R in the glomerular deposits shown by the arrows. PLA2R: phospholipase A2 receptor

He was initially treated with a transfusion of one unit of packed red blood cell (PRBC) and placed on epoetin alfa. Prednisone 60 mg daily was started for presumed acute interstitial nephritis and was continued for 11 days, 40 mg was administered on the 12th day, and 20 mg was continued for the next 10 days. His hypertension was managed with amlodipine, clonidine, and labetalol. A kidney biopsy was consistent with membranous glomerulonephritis with moderate segmental sclerosis, as well as tubulointerstitial nephritis (Figures 1, 2). Positive staining for PLA2R was noted in the glomerular deposits (Figure 3). These findings were consistent with primary MN. Treatment with rituximab was started and hemodialysis was initiated for azotemia. He was discharged after 13 days of hospitalization and completed four weekly doses of rituximab as an outpatient. He required hemodialysis initially after hospital discharge; however, it was discontinued after a month as his renal function normalized.

## Discussion

MN is a disease characterized by immune complex deposition on subepithelial glomerular capillaries [3]. Primary or idiopathic MN accounts for about two-thirds of cases. Secondary causes of MN include autoimmune diseases, medications, malignancies, and infections [2]. Overall, 70-80% of patients with primary MN are found to have PLA2R1 antibodies [3]. A smaller percentage of patients with secondary MN have anti-PLA2R1 antibodies as well [4].

MN has been reported following vaccine administration [1]. Cases of glomerular diseases have been reported following influenza vaccination, including a case of MN and acute interstitial nephritis. One patient presented with massive proteinuria and another with acute kidney injury (AKI) after receiving the 2009 H1N1 influenza vaccine [5].

We found several articles related to the COVID-19 vaccine and glomerulonephritis published since July 2021. In a study conducted at Mayo Clinic, Rochester, 13 cases of glomerulonephritis were found post-vaccination with mRNA COVID vaccine [6]. Among 13 patients, eight were newly diagnosed with glomerulonephritis, and five patients had relapsed. The median age was 62 years. A total of 12 patients out of the 13 were white and nine were male. Autoimmune disease was the most prevalent underlying disease, followed by cancer. Immunoglobulin G glomerulonephritis was the most common glomerulonephritis, and membranous glomerulonephritis was the second most common. Approximately 54% of patients received the Moderna vaccine and the rest received the Pfizer vaccine. Most patients presented after the second dose (10 out of 13, 77%), and the rest (3 out of 13) were reported after the first dose. Among the newly diagnosed glomerulonephritis, no PLA2R-positive MN cases were found. However, among the five relapsed glomerulonephritis cases, two had underlying PLA2R-associated MN [6]. In our case, the patient was a 56-year-old African American without any autoimmune comorbidity or history of cancer and was found to have PLA2R1-positive MN four weeks after receiving the first dose of the Moderna vaccine. It is unclear whether he was a newly diagnosed case of MN or a case of relapse. The presence of PLA2R1 antibodies suggests primary MN. It is also unclear whether his occupational exposure to formaldehyde and ethanol could have been a factor as toxic formaldehyde exposure has been associated with MN [7]; however, he had worked as a mortician for over 30 years without any incident and had not had any specific toxic exposure.

Pfizer COVID-19 vaccination has previously been associated with renal pathology in some patients [4]. Two healthy individuals who presented with macroscopic hematuria shortly after receiving a COVID-19 vaccination were subsequently diagnosed with immunoglobulin A nephropathy in one patient and crescentic glomerulonephritis in the other [8]. A case of minimal change disease (MCD) with nephrotic syndrome and AKI after the Pfizer-BioNTech COVID-19 vaccination has also been reported [9]. A kidney biopsy was performed for that patient and the findings were consistent with MCD [9]. Fourteen SARS-CoV-2-infected patients underwent kidney biopsy, of whom two were found to have anti-PLA2R-positive MN [10].

## Conclusions

Our case adds to the literature of cases with PLA2R-positive MN after receiving the COVID-19 vaccine. However, we cannot suggest or prove an association yet. We postulate that the vaccine may involve a loss of tolerance to the PLA2R antigen. In our case, the patient developed symptoms of viral infections that worsened after receiving the vaccine. Either the vaccine or the viral infection itself triggered the MN. Close follow-up of patients for worsening renal function or evidence of MN is warranted after COVID-19 vaccination. Further placebo-controlled studies of vaccinated patients are needed to determine whether SARS-CoV-2 virus vaccination is associated with a higher risk of MN and to identify potential predisposing factors and mechanisms of kidney injury in patients in whom it occurs.
